# Supplementary material for: Optical dopamine monitoring with dLight1 reveals mesolimbic phenotypes in a mouse model of neurofibromatosis type 1
Source: eLife. 2019 Sep 23;8:e48983. doi: 10.7554/eLife.48983 (PMC6819083; doi:10.7554/eLife.48983)
Supplement: Supplementary file 1. [file elife-48983-supp1.docx]

**Supplementary file 1. Trial-by-trial correlations between peak dLight1.2 responses to CS presentation and behavioral measures.**

| ***Nf1*^+/+^** | | | | ***Nf1*^+/-^** | | | |
| --- | --- | --- | --- | --- | --- | --- | --- |
| **Behavior** | **Trial** | **r** | **p** | **Behavior** | **Trial** | **r** | **p** |
| **CS: Acquisition** | 1 | -0.439 | 0.412 | **CS: Acquisition** | 1 | -0.632 | 0.028 |
| **CS: Acquisition** | 2 | -0.483 | 0.112 | **CS: Acquisition** | 2 | -0.397 | 0.201 |
| **CS: Acquisition** | 3 | -0.570 | 0.960 | **CS: Acquisition** | 3 | -0.310 | 0.326 |
| **CS: Acquisition** | 4 | -0.046 | 0.217 | **CS: Acquisition** | 4 | -0.416 | 0.178 |
| **CS: Acquisition** | 5 | 0.552 | 0.144 | **CS: Acquisition** | 5 | -0.380 | 0.223 |
| **CS: Acquisition** | 6 | 0.008 | 0.156 | **CS: Acquisition** | 6 | -0.495 | 0.102 |
| **CS: Acquisition** | 7 | -0.224 | 0.211 | **CS: Acquisition** | 7 | -0.081 | 0.803 |
| **CS: Acquisition** | 8 | 0.186 | 0.966 | **CS: Acquisition** | 8 | -0.315 | 0.318 |
| **CS: Acquisition** | 9 | -0.106 | 0.092 | **CS: Acquisition** | 9 | -0.219 | 0.494 |
| **CS: Acquisition** | 10 | -0.007 | 0.285 | **CS: Acquisition** | 10 | 0.184 | 0.567 |
| **CS: Acquisition** | 11 | 0.286 | 0.972 | **CS: Acquisition** | 11 | -0.200 | 0.534 |
| **CS: Acquisition** | 12 | 0.240 | 0.999 | **CS: Acquisition** | 12 | 0.014 | 0.965 |
| **CS: Acquisition** | 13 | 0.203 | 0.596 | **CS: Acquisition** | 13 | -0.157 | 0.625 |
| **CS: Acquisition** | 14 | 0.118 | 0.574 | **CS: Acquisition** | 14 | -0.049 | 0.879 |
| **CS: Acquisition** | 15 | 0.257 | 0.226 | **CS: Acquisition** | 15 | 0.214 | 0.504 |
| **CS: Freezing** | 1 | -0.387 | 0.169 | **CS: Freezing** | 1 | -0.641 | 0.025 |
| **CS: Freezing** | 2 | -0.096 | 0.965 | **CS: Freezing** | 2 | -0.248 | 0.437 |
| **CS: Freezing** | 3 | -0.414 | 0.956 | **CS: Freezing** | 3 | -0.298 | 0.347 |
| **CS: Freezing** | 4 | -0.305 | 0.657 | **CS: Freezing** | 4 | -0.396 | 0.203 |
| **CS: Freezing** | 5 | 0.382 | 0.222 | **CS: Freezing** | 5 | -0.220 | 0.492 |
| **CS: Freezing** | 6 | -0.070 | 0.485 | **CS: Freezing** | 6 | -0.437 | 0.156 |
| **CS: Freezing** | 7 | -0.664 | 0.261 | **CS: Freezing** | 7 | 0.002 | 0.996 |
| **CS: Freezing** | 8 | -0.106 | 0.582 | **CS: Freezing** | 8 | -0.304 | 0.337 |
| **CS: Freezing** | 9 | -0.433 | 0.710 | **CS: Freezing** | 9 | -0.001 | 0.997 |
| **CS: Freezing** | 10 | -0.332 | 0.580 | **CS: Freezing** | 10 | 0.180 | 0.575 |
| **CS: Freezing** | 11 | 0.169 | 0.200 | **CS: Freezing** | 11 | 0.073 | 0.821 |
| **CS: Freezing** | 12 | -0.042 | 0.659 | **CS: Freezing** | 12 | 0.042 | 0.896 |
| **CS: Freezing** | 13 | -0.322 | 0.821 | **CS: Freezing** | 13 | 0.335 | 0.288 |
| **CS: Freezing** | 14 | -0.053 | 0.907 | **CS: Freezing** | 14 | 0.308 | 0.330 |
| **CS: Freezing** | 15 | -0.137 | 0.822 | **CS: Freezing** | 15 | -0.061 | 0.850 |
| **CS: Latency** | 1 | 0.391 | 0.200 | **CS: Latency** | 1 | 0.584 | 0.046 |
| **CS: Latency** | 2 | 0.141 | 0.873 | **CS: Latency** | 2 | 0.151 | 0.640 |
| **CS: Latency** | 3 | 0.494 | 0.741 | **CS: Latency** | 3 | 0.177 | 0.583 |
| **CS: Latency** | 4 | 0.206 | 0.409 | **CS: Latency** | 4 | 0.272 | 0.392 |
| **CS: Latency** | 5 | -0.545 | 0.146 | **CS: Latency** | 5 | 0.118 | 0.714 |
| **CS: Latency** | 6 | -0.013 | 0.450 | **CS: Latency** | 6 | 0.295 | 0.351 |
| **CS: Latency** | 7 | 0.571 | 0.475 | **CS: Latency** | 7 | -0.167 | 0.603 |
| **CS: Latency** | 8 | 0.053 | 0.438 | **CS: Latency** | 8 | 0.180 | 0.576 |
| **CS: Latency** | 9 | 0.393 | 0.618 | **CS: Latency** | 9 | -0.072 | 0.823 |
| **CS: Latency** | 10 | 0.244 | 0.687 | **CS: Latency** | 10 | -0.220 | 0.492 |
| **CS: Latency** | 11 | -0.229 | 0.386 | **CS: Latency** | 11 | -0.234 | 0.464 |
| **CS: Latency** | 12 | -0.078 | 0.803 | **CS: Latency** | 12 | -0.185 | 0.566 |
| **CS: Latency** | 13 | 0.170 | 0.783 | **CS: Latency** | 13 | -0.468 | 0.125 |
| **CS: Latency** | 14 | 0.032 | 0.987 | **CS: Latency** | 14 | -0.409 | 0.186 |
| **CS: Latency** | 15 | 0.008 | 0.666 | **CS: Latency** | 15 | -0.003 | 0.993 |
