# Supplementary material for: Optical dopamine monitoring with dLight1 reveals mesolimbic phenotypes in a mouse model of neurofibromatosis type 1
Source: eLife. 2019 Sep 23;8:e48983. doi: 10.7554/eLife.48983 (PMC6819083; doi:10.7554/eLife.48983)
Supplement: Supplementary file 2. [file elife-48983-supp2.docx]

**Supplementary file 4. Correlations between dLight1.2 responses and behavioral measures.**

| ***Nf1*^+/+^** | | | | | ***Nf1*^+/+^** | | | |
| --- | --- | --- | --- | --- | --- | --- | --- | --- |
| **Feature** | **Behavior** | **r** | **p** | **Feature** | | **Behavior** | **r** | **p** |
| **CS Trial 1** | Acquisition | -0.293 | 0.412 | **CS Trial 1** | | Acquisition | -0.632 | 0.028 |
| **US Trial 1** | Acquisition | -0.292 | 0.413 | **US Trial 1** | | Acquisition | -0.146 | 0.650 |
| **US Anti-Peak Trial 1** | Acquisition | -0.387 | 0.269 | **US Anti-Peak Trial 1** | | Acquisition | 0.005 | 0.987 |
| **US Rebound Trial 1** | Acquisition | 0.043 | 0.906 | **US Rebound Trial 1** | | Acquisition | -0.091 | 0.779 |
| **US Rebound AUC Trial 1** | Acquisition | 0.209 | 0.562 | **US Rebound AUC Trial 1** | | Acquisition | 0.002 | 0.995 |
| **CS Trial 1** | Freezing | -0.471 | 0.169 | **CS Trial 1** | | Freezing | -0.641 | 0.025 |
| **US Trial 1** | Freezing | -0.396 | 0.257 | **US Trial 1** | | Freezing | -0.122 | 0.705 |
| **US Anti-Peak Trial 1** | Freezing | -0.761 | 0.011 | **US Anti-Peak Trial 1** | | Freezing | 0.320 | 0.311 |
| **US Rebound Trial 1** | Freezing | -0.153 | 0.674 | **US Rebound Trial 1** | | Freezing | -0.084 | 0.795 |
| **US Rebound AUC Trial 1** | Freezing | -0.223 | 0.536 | **US Rebound AUC Trial 1** | | Freezing | 0.109 | 0.736 |
| **CS Trial 1** | Latency | 0.443 | 0.200 | **CS Trial 1** | | Latency | 0.584 | 0.046 |
| **US Trial 1** | Latency | 0.352 | 0.319 | **US Trial 1** | | Latency | 0.123 | 0.704 |
| **US Anti-Peak Trial 1** | Latency | 0.680 | 0.030 | **US Anti-Peak Trial 1** | | Latency | -0.313 | 0.322 |
| **US Rebound Trial 1** | Latency | 0.102 | 0.780 | **US Rebound Trial 1** | | Latency | -0.025 | 0.938 |
| **US Rebound AUC Trial 1** | Latency | 0.080 | 0.826 | **US Rebound AUC Trial 1** | | Latency | -0.204 | 0.525 |
| **CS All Trials** | Acquisition | -0.553 | 0.097 | **CS All Trials** | | Acquisition | -0.368 | 0.239 |
| **US All Trials** | Acquisition | -0.173 | 0.633 | **US All Trials** | | Acquisition | -0.414 | 0.181 |
| **US Anti-Peak All Trials** | Acquisition | -0.086 | 0.814 | **US Anti-Peak All Trials** | | Acquisition | 0.352 | 0.261 |
| **US Rebound All Trials** | Acquisition | 0.277 | 0.438 | **US Rebound All Trials** | | Acquisition | 0.001 | 0.998 |
| **US Rebound AUC All Trials** | Acquisition | 0.283 | 0.429 | **US Rebound AUC All Trials** | | Acquisition | 0.198 | 0.537 |
| **CS All Trials** | Freezing | -0.420 | 0.227 | **CS All Trials** | | Freezing | -0.233 | 0.466 |
| **US All Trials** | Freezing | -0.220 | 0.541 | **US All Trials** | | Freezing | -0.249 | 0.435 |
| **US Anti-Peak All Trials** | Freezing | -0.378 | 0.281 | **US Anti-Peak All Trials** | | Freezing | 0.522 | 0.081 |
| **US Rebound All Trials** | Freezing | -0.126 | 0.728 | **US Rebound All Trials** | | Freezing | 0.421 | 0.173 |
| **US Rebound AUC All Trials** | Freezing | -0.128 | 0.725 | **US Rebound AUC All Trials** | | Freezing | 0.556 | 0.060 |
| **CS All Trials** | Latency | 0.451 | 0.191 | **CS All Trials** | | Latency | 0.092 | 0.776 |
| **US All Trials** | Latency | 0.171 | 0.636 | **US All Trials** | | Latency | 0.243 | 0.447 |
| **US Anti-Peak All Trials** | Latency | 0.340 | 0.336 | **US Anti-Peak All Trials** | | Latency | -0.468 | 0.125 |
| **US Rebound All Trials** | Latency | -0.060 | 0.870 | **US Rebound All Trials** | | Latency | -0.538 | 0.071 |
| **US Rebound AUC All Trials** | Latency | 0.007 | 0.986 | **US Rebound AUC All Trials** | | Latency | -0.674 | 0.016 |
