# Supplementary material for: Optical dopamine monitoring with dLight1 reveals mesolimbic phenotypes in a mouse model of neurofibromatosis type 1
Source: eLife. 2019 Sep 23;8:e48983. doi: 10.7554/eLife.48983 (PMC6819083; doi:10.7554/eLife.48983)
Supplement: Transparent reporting form [file elife-48983-transrepform.pdf]

eLIFE
Westbrook Centre, Milton Road
Cambridge CB4 1YG
UK
P 01223 855340
W elifesciences.org
T @elife

eLife's transparent reporting form

We encourage authors to provide detailed information *within their submission* to facilitate the interpretation and replication of experiments. Authors can upload supporting documentation to indicate the use of appropriate reporting guidelines for health-related research (see EQUATOR Network), life science research (see the BioSharing Information Resource), or the ARRIVE guidelines for reporting work involving animal research. Where applicable, authors should refer to any relevant reporting standards documents in this form.

If you have any questions, please consult our Journal Policies and/or contact us: editorial@elifesciences.org.

Sample-size estimation

- You should state whether an appropriate sample size was computed when the study was being designed
- You should state the statistical method of sample size computation and any required assumptions
- If no explicit power analysis was used, you should describe how you decided what sample (replicate) size (number) to use

Please outline where this information can be found within the submission (e.g., sections or figure legends), or explain why this information doesn't apply to your submission:

Sample sizes are clearly stated in the figure captions and in the Supplemental Statistical Analysis file, which provides details on all statistical tests performed in the manuscript. For all graphical representations of data in the manuscript, we have plotted all underlying individual data values so that that readers can more accurately assess the work. Additionally, we have provided information about how sample sizes were determined in the Statistical Analysis section of the Materials and Methods.

Replicates

- You should report how often each experiment was performed
- You should include a definition of biological versus technical replication
- The data obtained should be provided and sufficient information should be provided to indicate the number of independent biological and/or technical replicates
- If you encountered any outliers, you should describe how these were handled
- Criteria for exclusion/inclusion of data should be clearly stated
- High-throughput sequence data should be uploaded before submission, with a private link for reviewers provided (these are available from both GEO and ArrayExpress)

Please outline where this information can be found within the submission (e.g., sections or figure legends), or explain why this information doesn't apply to your submission:

eLife Sciences Publications, Ltd is a limited liability non-profit non-stock corporation incorporated in the State of Delaware, USA, with company number 5030732, and is registered in the UK with company number FC030576 and branch number BR015634 at the address Westbrook Centre, Milton Road Cambridge CB4 1YG, UK | March 2019

We have stated how data was replicated for each experiment in the associated methods section for each technique or in the figure caption. For example, we state that all behavioral tests were performed with at least two cohorts to minimize batch effects. For electrophysiology, individual neurons were recorded from slices prepared from at least three mice per genotype on different experimental days. For histological analysis, data was averaged per mouse unless otherwise noted. No outliers were removed during analysis, as stated in the Statistical Analysis section of the Methods. As outlined in the Materials and Methods, mice were only excluded from analysis if there were health concerns from the veterinary staff; photometry data was only excluded if the optical fiber was outside the target region (only one mouse) or if there was no dynamic signal two weeks after surgery; electrophysiological data was only discarded if neurons did not meet pre-determined criteria for cell health and recording quality.

Statistical reporting

- Statistical analysis methods should be described and justified
- Raw data should be presented in figures whenever informative to do so (typically when N per group is less than 10)
- For each experiment, you should identify the statistical tests used, exact values of N, definitions of center, methods of multiple test correction, and dispersion and precision measures (e.g., mean, median, SD, SEM, confidence intervals; and, for the major substantive results, a measure of effect size (e.g., Pearson's r, Cohen's d)
- Report exact p-values wherever possible alongside the summary statistics and 95% confidence intervals. These should be reported for all key questions and not only when the p-value is less than 0.05.

Please outline where this information can be found within the submission (e.g., sections or figure legends), or explain why this information doesn't apply to your submission:

As stated above, all relevant information regarding statistical tests is detailed in both the figure caption and in the Supplementary Statistical Analysis file. Exact p-values or 95% confidence intervals were stated throughout.

(For large datasets, or papers with a very large number of statistical tests, you may upload a single table file with tests, Ns, etc., with reference to sections in the manuscript.)

Group allocation

- Indicate how samples were allocated into experimental groups (in the case of clinical studies, please specify allocation to treatment method); if randomization was used, please also state if restricted randomization was applied
- Indicate if masking was used during group allocation, data collection and/or data analysis

Please outline where this information can be found within the submission (e.g., sections or figure legends), or explain why this information doesn't apply to your submission:

Mice were allocated to groups by an un-blinded independent lab member so that all investigators would remain blind to genotype throughout testing and analysis. Because littermate controls were used throughout, mice were allocated to experimental groups based on age and litter, as stated in the Experimental Animals section of the Materials and Methods.

Additional data files ("source data")

- We encourage you to upload relevant additional data files, such as numerical data that are represented as a graph in a figure, or as a summary table
- Where provided, these should be in the most useful format, and they can be uploaded as "Source data" files linked to a main figure or table
- Include model definition files including the full list of parameters used
- Include code used for data analysis (e.g., R, MatLab)
- Avoid stating that data files are "available upon request"

Please indicate the figures or tables for which source data files have been provided:

Viral vector plasmids used in this study are available on Addgene at http://www.addgene.org/Viviana_Gradinaru/. Codes used for fiber photometry signal extraction and analysis are available at https://github.com/GradinaruLab/dLight1. Source data is available at www.doi.org/10.7303/syn18904024.

eLife Sciences Publications, Ltd is a limited liability non-profit non-stock corporation incorporated in the State of Delaware, USA, with company number 5030732, and is registered in the UK with company number FC030576 and branch number BR015634 at the address Westbrook Centre, Milton Road Cambridge CB4 1YG, UK | March 2019
